# Criticality and chaos in auditory and vestibular sensing

**DOI:** 10.1038/s41598-024-63696-3

**Published:** 2024-06-06

**Authors:** Justin Faber, Dolores Bozovic

**Affiliations:** 1 Department of Physics and Astronomy, University of California, Los Angeles, CA 90095 USA; 2 California NanoSystems Institute, University of California, Los Angeles, CA 90095 USA

**Keywords:** Nonlinear phenomena, Phase transitions and critical phenomena, Biophysics, Biological physics

## Abstract

The auditory and vestibular systems exhibit remarkable sensitivity of detection, responding to deflections on the order of angstroms, even in the presence of biological noise. The auditory system exhibits high temporal acuity and frequency selectivity, allowing us to make sense of the acoustic world around us. As the acoustic signals of interest span many orders of magnitude in both amplitude and frequency, this system relies heavily on nonlinearities and power-law scaling. The vestibular system, which detects ground-borne vibrations and creates the sense of balance, exhibits highly sensitive, broadband detection. It likewise requires high temporal acuity so as to allow us to maintain balance while in motion. The behavior of these sensory systems has been extensively studied in the context of dynamical systems theory, with many empirical phenomena described by critical dynamics. Other phenomena have been explained by systems in the chaotic regime, where weak perturbations drastically impact the future state of the system. Using a Hopf oscillator as a simple numerical model for a sensory element in these systems, we explore the intersection of the two types of dynamical phenomena. We identify the relative tradeoffs between different detection metrics, and propose that, for both types of sensory systems, the instabilities giving rise to chaotic dynamics improve signal detection.

## Introduction

Auditory and vestibular systems perform tasks that are crucial for the survival of an animal, enabling it to navigate in space, detect signals from predators or prey, identify and attract potential mates, and communicate with members of the same species. To achieve these tasks, the sensory system must detect extremely weak signals, extract them from noisy environments, distinguish tones of closely spaced frequencies, and precisely parse temporal information. Specifically, near our threshold of hearing, we are able to detect displacements of the eardrum smaller than the width of the hydrogen atom^1^. This detection occurs in the presence of internal thermal fluctuations of equal or higher magnitude. Further, the temporal resolution of humans typically reaches 10 ms^2,3^, enabling sound localization through interaural time differences^4^. Finally, we are able to resolve tones that differ in frequency by only a fraction of a percent^5^. In parallel, the auditory system achieves immense dynamic range in both amplitude and frequency of acoustic detection. We are able to detect sounds that span over 12 orders of magnitude in intensity and 3 orders of magnitude in frequency. These broad ranges are reflected in our logarithmic decibel scale for sound intensity and logarithmic spacing of musical intervals.

The remarkable features of the auditory system rely heavily on two factors: active amplification and nonlinear response. First, the system has been shown to violate the fluctuation dissipation theorem, indicating that it cannot be governed by equilibrium statistical mechanics^6^. Vast amounts of empirical evidence obtained in vivo demonstrate that the inner ear expends energy to amplify signals^7^. Second, compressive nonlinearities in the response to external stimuli enable the extensive dynamic range, while maintaining sensitivity to weak signals^6,8^. These nonlinearities have been detected at all scales measured, from individual sensory cells to in vivo phenomena known as phantom tones^9–11^. How the auditory system utilizes energy expenditure and nonlinearities to achieve its remarkable detection characteristics, however, remains unknown after more than 7 decades of research^12–14^.

The theoretical framework for auditory detection that was developed over the past 20 years is based on the notion of a dynamical system poised near criticality^15^, on the verge of autonomous oscillation. The models apply the normal form equation for the supercritical Hopf bifurcation to describe the auditory system, and elegantly capture the mechanical sensitivity, frequency selectivity, and amplitude-compressive response, thus reproducing a broad range of experimental results^16,17^. In the vicinity of the bifurcation, the system’s sensitivity to external signals increases, while the frequency selectivity sharpens, thus pointing to criticality as the optimal regime for signal detection^18^.

However, while proximity to a bifurcation yields many advantages, the description contains an innate constraint: at criticality, the system becomes infinitely slow, with transient times diverging as a result of *critical slowing down*. This sluggish behavior poses an undesirable trade-off between the sensitivity of a detector and its speed, and is inconsistent with the high temporal acuity exhibited by our auditory system. Furthermore, at the critical point, the system is very sensitive to the effects of stochastic fluctuations, which limits some of the advantages observed in the deterministic models. As noise is a ubiquitous component of any biological system, and its effects specifically near criticality are not negligible, this constraint limits the advantages of tuning a system near a bifurcation point.

An alternate theoretical framework, developed to reconcile the requirement for high sensitivity of detection with the need for a rapid temporal response, is based on the notion of chaotic dynamics^19^. When a system is poised in a chaotic regime, even infinitesimally weak external perturbations trigger large changes in the subsequent trajectory, thus yielding high sensitivity. As a result of this exponential divergence, the system also responds and resets rapidly. Hence, a chaotic system avoids the inherent tradeoff observed with criticality.

In a prior study, we demonstrated experimentally the presence of a chaotic attractor in the innate and driven oscillations exhibited by sensory hair cells in vitro^20^. Using mathematical methods from dynamical systems literature, we confirmed that the oscillator contains a deterministic component and is not completely dominated by biological noise and other stochastic processes. These mathematical methods have also been used to show the presence of chaos in experimental recordings of human otoacoustic emissions^21^.

We note that chaos is typically considered a harmful element in applied mathematics literature, as it limits control and predictability. However, it has been proposed to play a potentially helpful role in certain biological systems, as it enhances their dynamical complexity^22,23^. Further, using a numerical model and experimental recordings, we showed chaos to be beneficial to individual hair cells, as the instabilities from which chaos arises enhance the sensitivity and temporal resolution of the response^19^. As chaotic regimes can arise in the presence of three or more degrees of freedom, we predict that many more examples of living systems utilizing chaotic dynamics will be uncovered in the future. This phenomenon is reminiscent of stochastic resonance, a mechanism exhibited by excitable systems, where signal detection is surprisingly improved with the addition of noise. Stochastic resonance has been shown to be present in the dynamics of Hopf oscillators^24^, as well as experimental measurements from the bullfrog inner ear^25^.

In the present work, we aim to compare the relative advantages of criticality versus the instabilities that cause chaos, in a theoretical description of auditory and vestibular detection. In particular, we explore the interface of these two theoretical models: a system poised near the supercritical Hopf bifurcation and one poised in the chaotic regime, as well as the continuum describing transitions between the regimes. To assess separately each of the key features exhibited by these sensory systems, we characterize the sensitivity, frequency selectivity, temporal acuity, and power-law amplitude response of a Hopf oscillator, near and far from criticality, in the presence and absence of chaos. As stochastic fluctuations play a non-negligible role in dynamical systems, we find the relative tradeoffs between these detection characteristics in the presence of noise. We then combine these independent metrics to propose a simple conceptual framework for auditory and vestibular detection.

## Hopf bifurcation and critical slowing down

The inner ear of vertebrates contains a number of end organs that specialize in either auditory or vestibular detection, the latter including both translational and rotational movement. While the pathways by which external signals reach the inner ear vary, they ultimately result in mechanical vibrations of internal structures; hence, the two sensory systems exhibit many features in common. Conversion of mechanical energy of a sound, vibration, or acceleration into electrical energy in the form of ionic currents is performed by specialized, sensory hair cells of both the auditory and vestibular systems. The hair cell gets its name from the rod-like, inter-connected stereocilia that protrude from the apical surface, which are collectively named the hair bundle. An incoming stimulus pivots the stereocilia and modulates the open probability of the transduction channels embedded in the tips of the stereocilia^26,27^. In several species, these hair bundles have been shown to exhibit active limit-cycle oscillations in the absence of applied stimulus^6,28–30^. Though the role of these spontaneous hair-bundle oscillations in vivo has yet to be established, they serve as an experimental probe for studying this active system, as they lead to sub-nanometer thresholds in vitro^31,32^.

The Hopf oscillator has been extensively used for modeling and understanding the phenomenon of active, spontaneous hair-bundle oscillations, as well as more global features of the auditory and vestibular systems^18,33^. When the system is poised on the verge of instability (near the Hopf bifurcation), it becomes extremely sensitive to small perturbations. In the absence of noise, the amplitude gain of the response diverges as the system approaches criticality. The model was shown to capture active amplification and power-law amplitude response observed both in vivo and in vitro on a number of different species^5^.

A Hopf oscillator is described by time-dependent complex variable, *z*(*t*), which is governed by a normal form equation, which in its simplest version, takes the form:

1 $$\begin{aligned} \frac{dz}{dt} = (\mu + i\omega _0)z - |z|^2z + F(t) + \eta (t) \end{aligned}$$

where $\mu$ and $\omega _0$ are the control parameter and characteristic frequency of the detector, respectively. *F*(*t*) represents the external forcing on the system, while $\eta (t)$ is a stochastic variable, representing thermal noise. This variable is complex, with independent real and imaginary parts, both of which have statistics of Gaussian white noise: $\langle \eta (t)\rangle = 0$, $\langle \eta (t)\eta (t')\rangle = 0$, and $\langle \eta (t)\bar{\eta }(t')\rangle = 4D\delta (t-t')$, where $\bar{\eta }$ is the complex conjugate of $\eta$, and *D* defines the noise strength.

In the absence of forcing and noise ($F(t) = D = 0$), the system can be more easily understood in polar coordinates, by letting $z(t) = r(t)e^{i\theta (t)}$, thereby separating the complex variable into two real variables. This results in the pair of equations,

2 $$\begin{aligned} \frac{dr}{dt} = \mu r - r^3 \quad \text{and} \quad \frac{d\theta }{dt} = \omega _0, \end{aligned}$$

which describe the amplitude and phase dynamics of the system, respectively. Notice that the instantaneous frequency $\frac{d\theta }{dt}$ is constant, having no dependence on the oscillator’s amplitude, *r*(*t*). This defines an isochronous oscillator.

The amplitude dynamics are determined by the control parameter, $\mu$. For $\mu < 0$, the system displays a stable fixed point with increasing local stability for more negative values of the control parameter. As the control parameter approaches the critical point at $\mu = 0$, the system loses its local stability at the origin, and stable limit-cycle oscillations emerge. For $\mu > 0$, as this parameter increases, the spontaneous oscillations grow larger, and the local stability of the limit cycle increases. Precisely at the critical point, the amplitude gain of the system diverges in the absence of noise, as an infinitesimal perturbation causes large-amplitude displacements^18^ (Fig. S1). For both positive and negative values of the control parameter, the amplitude returns to its steady-state exponentially, with characteristic time scale proportional to $1/\mu$ (see Supplementary Material). In the vicinity of the bifurcation, the linear term becomes vanishingly small, and the system returns to steady-state very slowly, with perturbations diminishing according to a power-law. As the control parameter approaches the Hopf bifurcation, this time scale diverges without bound at $\mu =0$, a dynamical systems phenomenon known as *critical slowing down* (Fig. S1).

**Figure 1:**
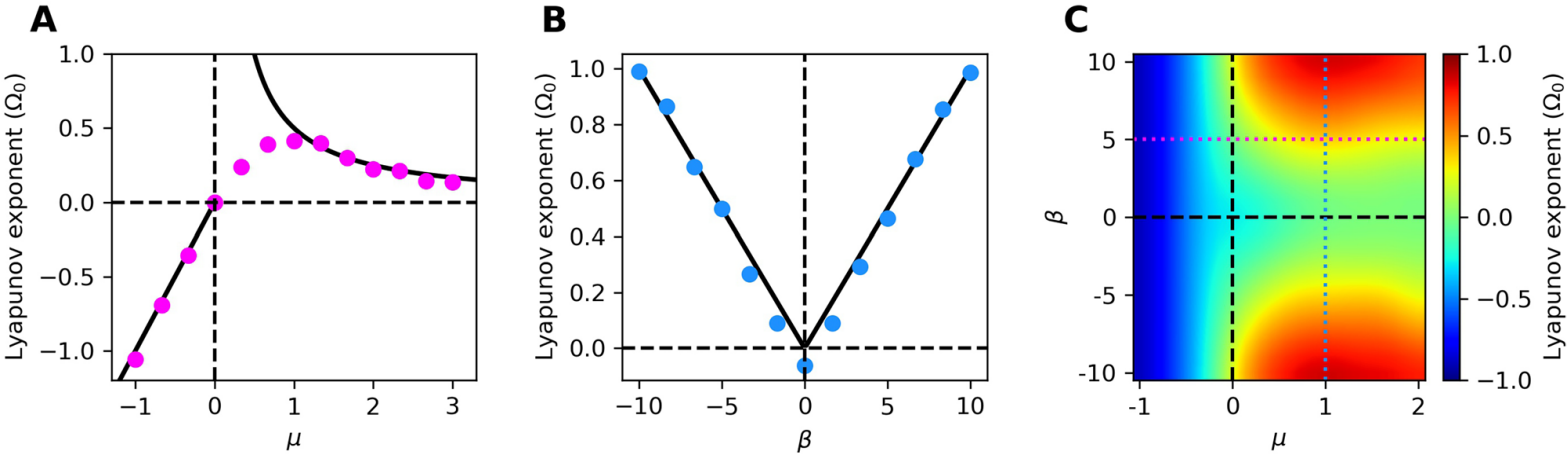
(**A**) Lyapunov exponent calculated numerically with $\beta =5$, for a range of control parameters. (**B**) Lyapunov exponent calculated numerically with $\mu =1$, for a range of degrees of nonisochronicity. For (**A, B**), the black curves correspond to the analytic approximations of the Lyapunov exponent. (**C**) Lyapunov exponent calculated numerically throughout the parameter space. The dotted lines correspond to the cross-sections plotted in (**A, B**). For all panels, $D=0.1$.

## Nonisochronicity and chaos

In several studies of auditory and vestibular systems, a more general version of the Hopf oscillator was considered^19,34^. The equation takes the form

3 $$\begin{aligned} \frac{dz}{dt} = (\mu + i\omega _0)z - (\alpha + i\beta )|z|^2z + F(t) + \eta (t) , \end{aligned}$$

where $\alpha$ and $\beta$ are introduced to characterize the nonlinearity of the system, while all the other parameters carry the same meaning as in the isochronous case previously described. In the absence of stimulus and noise ($F(t) = D = 0$), this equation can be written in polar coordinates as

4 $$\begin{aligned} \frac{dr}{dt} = \mu r - \alpha r^3 \quad \text{and} \quad \frac{d\theta }{dt} = \omega _0 - \beta r^2 \end{aligned}$$

For $\mu > 0$, a stable limit cycle exists at radius $r_0 = \sqrt{\frac{\mu }{\alpha }}$. The frequency at this limit cycle is $\Omega _0 = \omega _0 - \beta r_0^2 = \omega _0 - \beta \mu / \alpha$, where $\omega _0$ is the frequency at the Hopf bifurcation. This more general description reduces to the traditional, isochronous form when $\alpha = 1$ and $\beta = 0$. We restrict our analysis to systems with $\alpha = 1$ and vary $\beta$ to control the level of nonisochronicity. This new parameter causes the frequency of oscillation to depend on the amplitude of oscillation. Experimental recordings obtained from in vitro preparations of hair cells have shown signatures of nonisochronicity in spontaneously oscillating hair bundles. The coupling between frequency and amplitude can be seen through experimental manipulations of the innate limit cycle, such as imposing large deflections on the hair bundle^35^, or by adjusting the calcium concentration of the surrounding solution^19^.

By coupling the radial and phase degrees of freedom, the presence of nonisochronicity ($\beta \ne 0$) introduces significant complexity to the dynamics of this system. It distorts the Lorentzian shape of the frequency response curve^36^ and can even produce branching of this curve, discontinuities, and bistability for a range of frequencies, as well as hysteretic behavior in response to frequency sweeps^37^. The presence of nonisochronicity also makes the system susceptible to chaotic dynamics. Chaos in dynamical systems is characterized by extreme sensitivity to initial conditions and exponential divergence of neighboring trajectories. In the nonisochronous Hopf oscillator, chaos can arise from sinusoidal or impulsive external forcing, as well as systems driven purely by stochastic white noise^19,38^.

The rate of divergence of neighboring trajectories is expected to follow $|\Delta z(t)| \propto e^{\lambda t}$, where $\Delta z(t)$ is the Euclidean distance between two nearby phase-space trajectories, and $\lambda$ is the Lyapunov exponent. Chaotic systems are often defined by $\lambda > 0$. For quiescent, non-chaotic systems, the Lyapunov exponent is negative and characterizes the system’s stability, or the rate at which the system returns to equilibrium following a perturbation. For a Hopf oscillator with $\mu < 0$ and no forcing or noise, the Lyapunov exponent can be found by expanding around the stable fixed point (see Supplementary Material). One finds that in this simple case, $\lambda = \mu$, which indicates that the system becomes more stable at the origin for more negative values of $\mu$.

In the absence of noise, as the control parameter crosses from the quiescent to the oscillatory regime, the Lyapunov exponent increases to zero and remains there even for ($\mu > 0$). Since perturbations in the phase neither diverge nor converge, trajectories are neutrally stable along the angular direction of the limit cycle. In the presence of noise, analytic approximations often becomes intractable, but numerical simulations yield values of the Lyapunov exponent that can be positive, negative, or zero.

For a noisy nonisochronous Hopf oscillator, an analytic approximation can be made in the regime of sufficiently stable limit cycles. In this regime, the Lyapunov exponent shows a simple dependence on both the control and nonisochronicity parameters^19^,

5 $$\begin{aligned} \lambda \approx \frac{|\beta |D}{\mu } . \end{aligned}$$

This approximation is particularly useful in weakly chaotic regimes, where numerical simulations are computationally expensive. We show the robustness of this approximation for sufficiently stable limit cycles, as well as the simpler approximation for quiescent systems (Fig. 1A-B). The analytic approximation however breaks down as the system approaches criticality from the oscillatory side, because the assumption of a sufficiently stable limit cycle is no longer valid. We therefore provide a map of the Lyapunov exponent calculated numerically throughout the parameter space (Fig. 1C). These calculations serve to show the level of chaos as the system crosses the Hopf bifurcation, a regime not explored analytically. Further, this mapping illustrates the connection between the Lyapunov exponent and the level of nonisochronicity. Note, however, that the level of chaos depends on the noise strength, *D*, and hence the exact map changes if the noise strength is varied. In the following sections, we assess the impact of the control parameter, $\mu$, the nonisochronicity parameter, $\beta$, and the noise strength, *D*.

**Figure 2:**
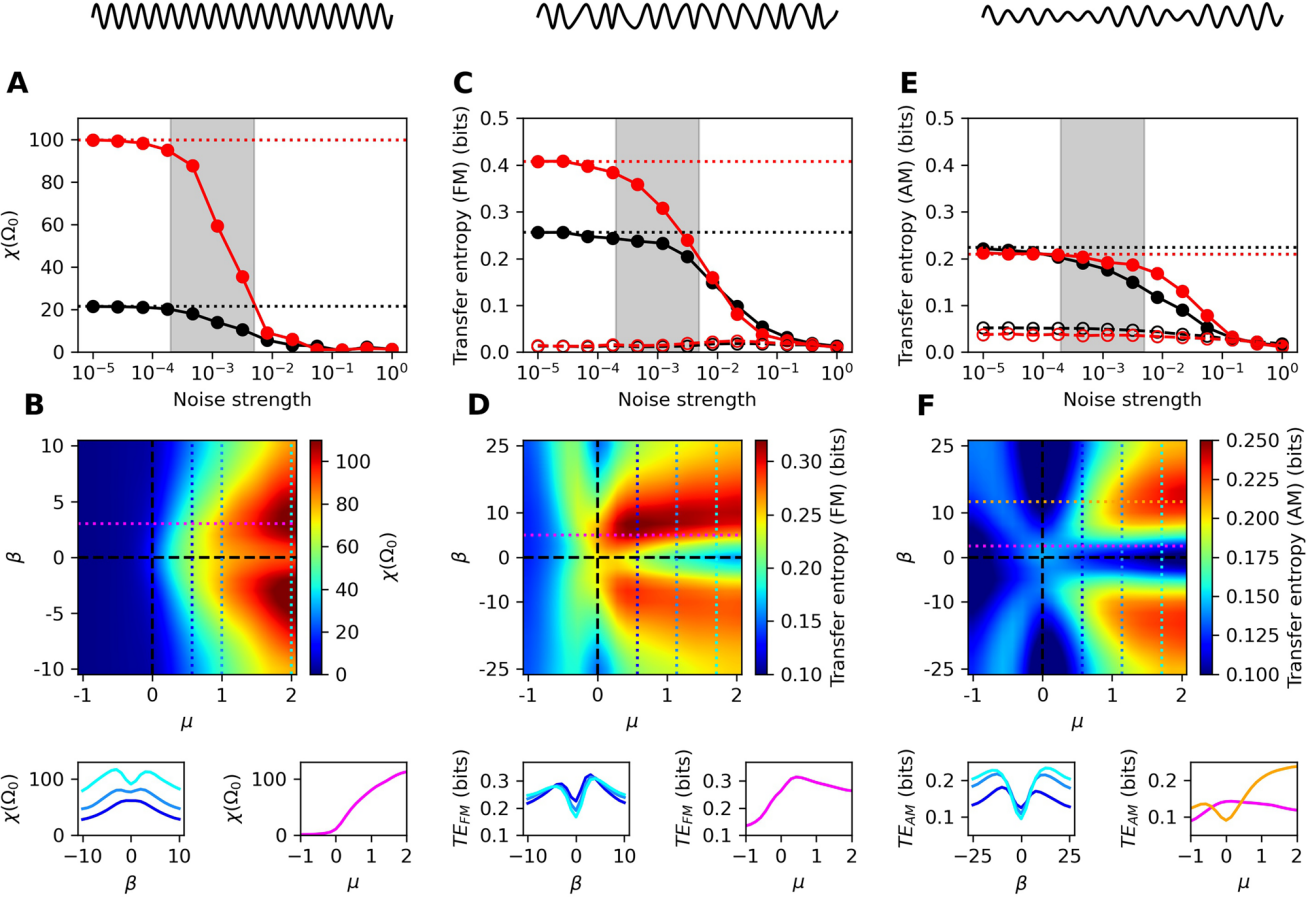
(**A, C, E**) Sensitivity metrics for the critical system ($\mu =\beta = 0$, black filled points) and chaotic system ($\mu =1, \beta = 5$, red filled points) for a range of noise strengths. Dotted horizontal lines correspond to the value of each metric in the deterministic limit. In (**C, E**), open points correspond to transfer entropy in the reverse direction (response to stimulus) and serve as controls. The grey, shaded regions estimate the biological noise level experienced by hair bundles (1–5% of the signal amplitude^35^). The three stimulus types are illustrated above each column: pure tone, frequency modulation (FM), and amplitude modulation (AM). (**B, D, F**) Heatmaps of the three measures throughout the parameter space with $D=10^{-3}$. Cross-sections along the colored, dotted lines are shown below.

**Figure 3:**
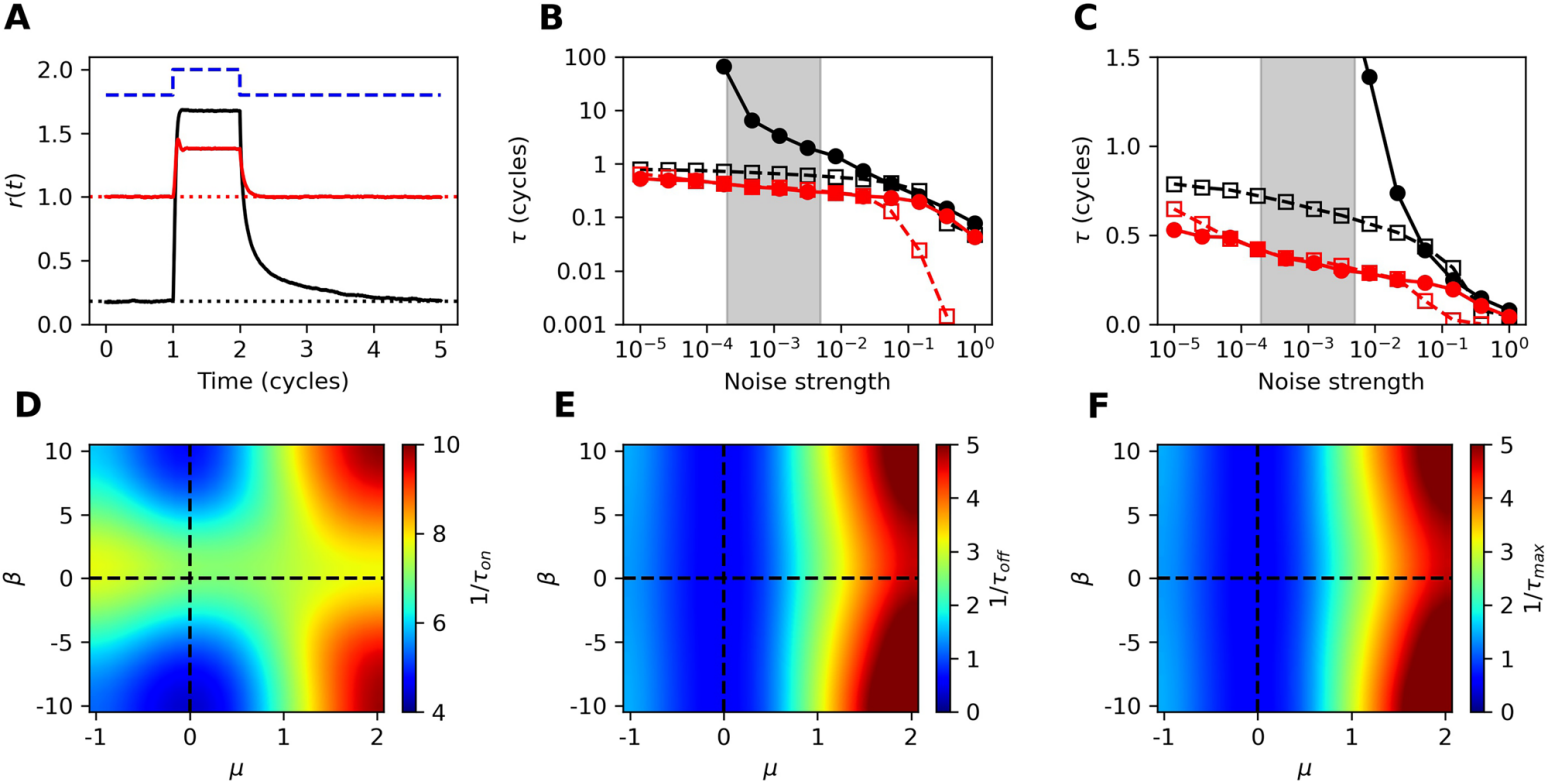
(**A**) Responses of the critical ($\mu =\beta = 0$, black curve) and chaotic ($\mu =1, \beta = 5$, red curve) systems to a step stimulus (illustrated by the blue dashed curve). Response curves represent averages over 64 simulations, each with different initial conditions and realizations of noise. The dotted lines indicate the mean amplitude prior to the step onset. (**B**) Response times (open squares) and return times (filled circles) of the critical (black) and chaotic (red) systems for several levels of noise. The return time of the critical system rapidly diverges as the noise is reduced. Return times longer than 100 cycles are not plotted. The grey, shaded regions estimate the biological noise level experienced by hair cells. (**C**) Same data as (**B**) but zoomed in and on a linear scale. (**D–F**) Heatmaps of the the speed of response, speed of return, and slower of the two measures. For every combination of parameters, the return time exceeded the response time ($\tau _{off} > \tau _{on}$). For all heatmaps, $D=10^{-3}$.

## Mechanical sensitivity and information transfer

Near the Hopf bifurcation, a noiseless system displays immense sensitivity, with the amplitude gain of the response diverging precisely at the bifurcation^18^. However, this compliance also makes the system susceptible to stochastic fluctuations. In the oscillatory regime, the detector becomes more resistant to noise, but also harder to entrain by external signals. Nonisochronicity can assist detectors in synchronizing to external signals; however, this effect can likewise increase susceptibility to external noise and off-resonance stimulus frequencies. We hence determine the sensitivity of the Hopf oscillator throughout the $\mu \beta$-plane and identify the preferred regimes for several types of stimulus.

We first consider a weak, single-tone, on-resonance stimulus in the presence of noise. We employ the linear response function, $\chi (\Omega _0)$, to characterize the sensitivity of the system (see Numerical Methods). For systems that exhibit autonomous oscillations, this measure exhibits a spurious non-zero value if the response does not synchronize to the signal. To avoid this issue, we ensure that only the phase-locked component is included in the calculation. We do this by averaging the responses over many systems, each prepared with different initial phases, thereby averaging out any oscillatory component that does not synchronize to the stimulus. It has previously been shown that the isochronous Hopf oscillator detects this signal best when poised as far into the oscillatory regime as possible^39^. Our results are consistent with this finding, with even further improvement when the system is weakly nonisochronous (Fig. 2). Further, we show that detectors in this regime outperform those in the critical regime for all levels of noise considered.

Next, we consider stochastic modulations in the amplitude (AM) and frequency (FM) of this signal (see Numerical Methods). Unlike the pure-tone stimulus, these signals carry information in their modulations. We therefore think of the detector not only as a mechanical resonator, but also as an information-theoretic receiver. We employ transfer entropy as the measure of information captured by the receiver^40^. This measure is particularly useful, as it carries no assumptions about which features of the external signal are important. Instead, it directly measures the amount of information transmitted from one process to another, and can even be used to establish causality between two processes. For all parameter ranges tested and for both types of modulation, detectors that are poised in the oscillatory, nonisochronous regime capture the most information from the applied stimulus. We further explored the sensitivity of this detection metric to stochastic fluctuations and found the nonisochronous regime to yield more robustness than the critical, isochronous regime (Fig. 2).

**Figure 4:**
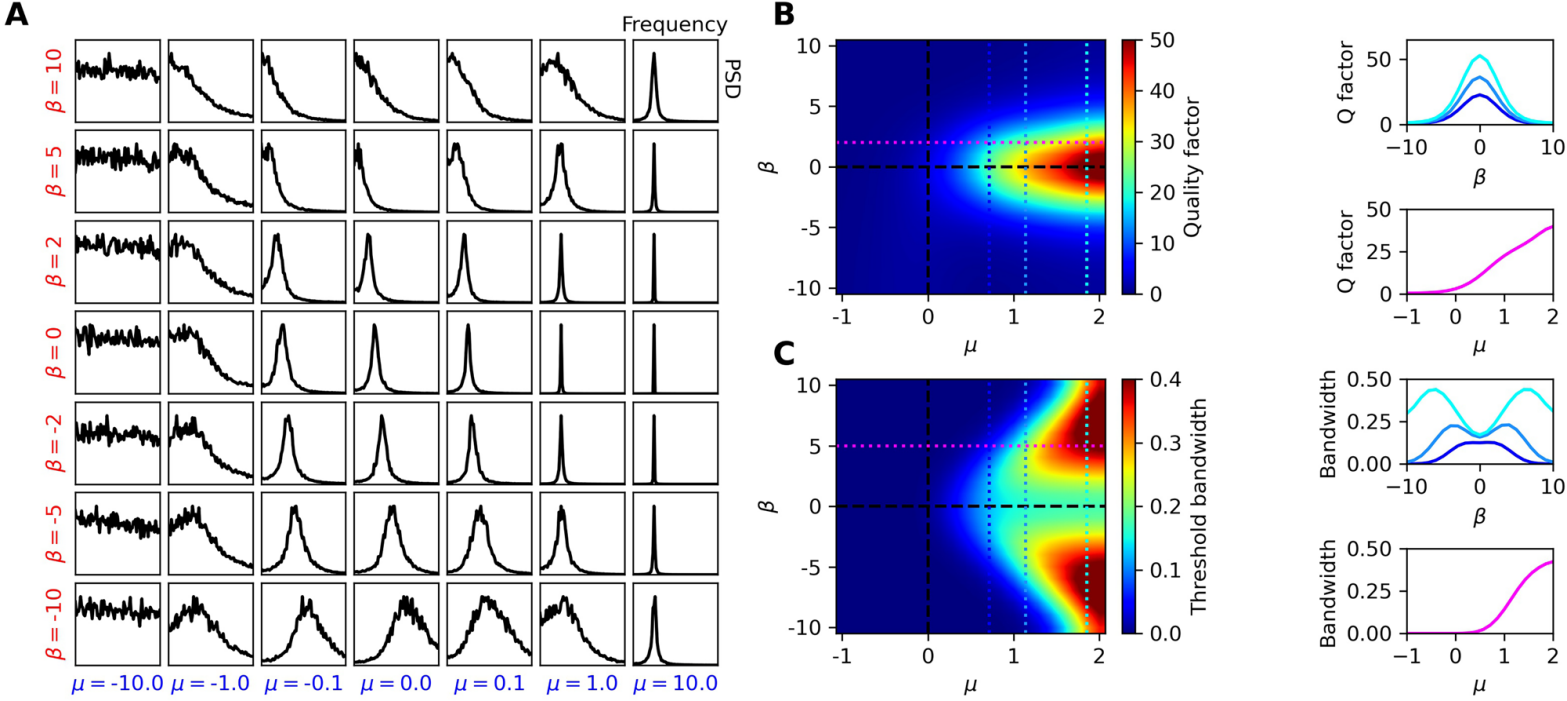
(**A**) Power spectral density of the system through the parameter space, in response to white noise stimulus. All curves are normalized to their peak values and plotted on a linear scale. (**B**) Heatmap of the quality factor as measured from the response curves. (**C**) Heatmap of the threshold bandwidth, indicating the frequency range for which the Fourier-component amplitudes of the response exceed 0.1. For all panels, $D=0.01$. Cross-sections of the heatmaps are shown on the right.

## Temporal acuity

High temporal acuity is essential for a sensory system to be responsive to brief signals. Further, localization of sound by vertebrates relies on interaural time differences as small as a few tens of microseconds, which correspond to temporal differences of just a fraction of a single stimulus cycle. When such small differences are biologically meaningful, the system’s response to a stimulus and its subsequent return to steady state must occur rapidly. To characterize this temporal acuity, we apply a step stimulus and measure the time the system requires to reach its steady-state response, as well as the time it takes to return to its unstimulated steady state after cessation of the signal.

We previously found that nonisochronicity greatly increases the speed of response to a step stimulus^38^. These results are consistent with experimental measurements that demonstrated a correlation between the speed of response and the level of chaos, quantified by the Kolmogorov entropy^19^. In the present work, we extend our analysis to variations in the control parameter and determine the temporal acuity of the Hopf oscillator in the $\mu \beta$-plane. We apply a step stimulus to the detector (Fig. 3A), averaging over many simulations with different initial conditions and realizations of noise. This method of calculating the mean response averages out the stochastic fluctuations, as well as any autonomous oscillations that would otherwise obscure slow modulations to the mean-field response.

We define the response time ($\tau _{on}$) to be the time it takes the system to settle to and remain within 4 standard deviations of its steady-state mean value following the onset of the step stimulus. Likewise, we define the return time ($\tau _{off}$) to be the time it takes the mean response of the system to become indistinguishable (within a standard deviation) from its value prior to the stimulus. We consider the limiting factor ($\tau _{max}$) to be the maximum of the two time constants. For all parameters tested, the return time was the limiting factor (Fig. 3D-F). For simplicity, we therefore drop the subscripts and let $\tau = \tau _{max} = \tau _{off}$.

Stochastic fluctuations not only obscure the mean-field response, but can also distort it. As the noise level increases, so does the average amplitude of the system. This increase in the baseline amplitude reduces the distance traveled by the returning mean response, effectively increasing the speed ($\frac{1}{\tau }$) according to our definition. We measure the speed of response over five orders of magnitude in noise strength and find that the oscillatory, nonisochronous system is faster to return to the baseline than the critical system (Fig. 3B-C). For extremely large levels of noise, the effects of critical slowing down are removed. However, the levels of noise sufficient to equalize the speed of the two types of systems are so large as to lead to a vast reduction in the sensitivity (Fig. 2A, C, and E).

**Figure 5:**
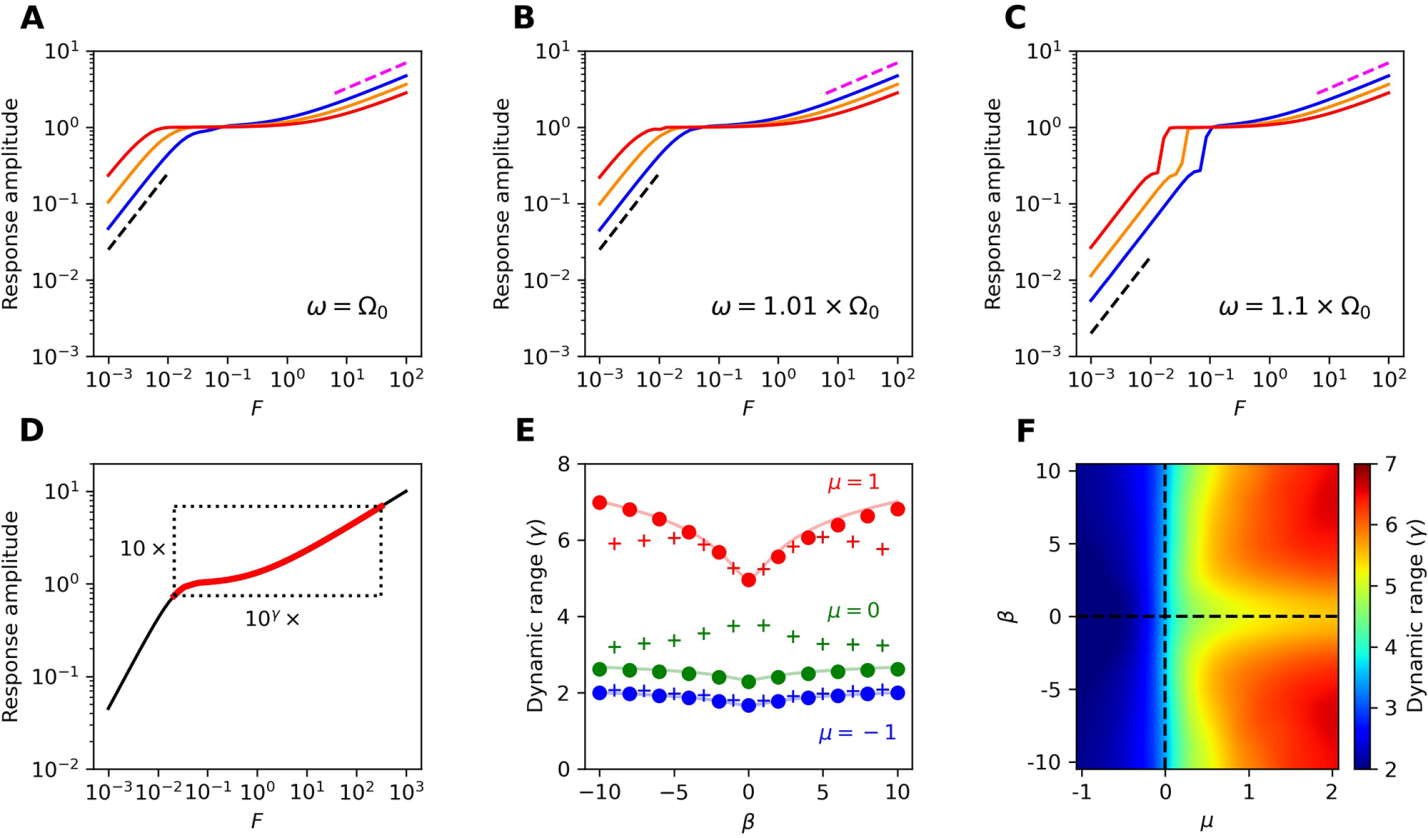
Response amplitude from a pure-tone stimulus for on-resonance (**A**) and detuned (**B, C**) stimulus frequencies as indicated in each panel. Blue, orange, and red curves correspond to $\beta =0$, 2, and 5, respectively. Black dashed lines indicate linear growth, while pink dashed lines indicate power-law growth with $|z(\omega )| \propto F^{\frac{1}{3}}$. For (**A–C**), $\mu =1$ and $D=0$. (**D**) Illustration of how the dynamic range, $\gamma$, is calculated. (**E**) Dynamic range as a function of $\beta$ for several fixed values of $\mu$. Solid curves correspond to the analytic calculation, while circles and crosses correspond to simulations with $D=0$ and $D=0.001$, respectively. (**F**) Heatmap of the dynamic range throughout parameter space, calculated from numerical simulations with $D=0.001$. For (**E, F**), we use a detuning of $\omega = 1.01\Omega_0$.

## Frequency selectivity and broadband detection

Although chaos can make dynamical systems sensitive to small perturbations and external signals, these systems tend to synchronize to a broad range of frequencies. This may be a beneficial effect for broadband detectors, such as vestibular systems, as it would increase the energy transmitted to the system and likely result in lower thresholds of detection. However, for frequency selective detectors, like those of auditory systems, this effect may be harmful.

We stimulate the Hopf oscillator with additive Gaussian white noise by adjusting *D* and measure the power spectrum of the response throughout the parameter space (Fig. 4A). As these curves indicate how sharply the system filters the white noise, we use them to characterize the frequency selectivity^41^. We adjust $\beta$ and $\omega _0$ together so as to keep the limit-cycle frequency fixed at $\Omega _0 = \omega _0 - \frac{\beta \mu }{\alpha } = 1$ in the deterministic limit. We then introduce stochastic white noise into the simulations and calculate the response curves. We observe that the frequency selectivity increases with increasing $\mu$ and decreasing $|\beta |$. Near the Hopf bifurcation, we see that the resonance frequency depends on $\beta$. This is a consequence of the noise altering the steady-state amplitude, and thereby the frequency, in this compliant regime.

To characterize the frequency selectivity of the Hopf oscillator, we employ the quality factor of the response by estimating the full width at half the maximum ($\Delta f$) of the response curves. The unitless quality factor is defined as $Q = \frac{f_0}{\Delta f}$, where $f_0$ is the peak frequency. As expected, the most frequency selective parameter regime occurs at high values of $\mu$ and low values of $|\beta |$ (Fig. 4B). The quality factor is useful for characterizing the frequency selectivity of a single-frequency or narrowband detector.

As some auditory and most vestibular systems are responsible for detecting a broader range of frequencies, we utilize the threshold bandwidth (*BW*) for characterizing multi-frequency or broadband detection, a metric suggested in^39^. We estimate this measure by taking the range of frequencies whose Fourier components have magnitudes exceeding a given threshold. We choose the threshold to be 0.1, which corresponds to approximately a factor of 10 above the noise floor of the critical system. The threshold bandwidth increases with increasing $\mu$, as the energy from the spontaneous oscillations amplifies the signal (Fig. 4C). Further, the threshold bandwidth initially increases with increasing $|\beta |$ due to the broadening of the frequency response curves. However, the *BW* then diminishes for very large values of $|\beta |$, as the energy becomes so spread out in frequency space that few components exceed the threshold.

## Power-law scaling of response

The human auditory system can detect a range of stimulus amplitudes spanning over 6 orders of magnitude in pressure. This process relies on nonlinearities in order to both amplify weak sounds and attenuate loud sounds, thereby protecting the system from damage. These nonlinearities have been measured in vivo through otoacoustic emissions and through laser measurements of basilar membrane motion. Further, the attenuation of large amplitudes has been observed in vitro on active hair bundles^16^. At weak forcing, hair cells display a linear response; as the stimulus amplitudes increase, the amplitude response scales as $F^\frac{1}{3}$. The linear and 1/3-power-law responses are reproduced well by the Hopf oscillator, as either the linear or the cubic term dominates in different regimes. Near criticality, the range over which the power law is observed increases.

We have demonstrated that the nonisochronicity parameter, $\beta$, improves the sensitivity to weak signals. We here show that this parameter also causes stronger attenuation of the response at large stimulus amplitudes. This increased attenuation can be understood by calculating the response in the strong-forcing limit, where the cubic term dominates (see Supplementary Material). In this limit, the response amplitude is scaled by $\frac{1}{(\alpha ^2 + \beta ^2)^\frac{1}{6}}$. The combination of these two effects yields an increase in the range of stimulus levels over which amplitude compression is observed and, hence, increases the dynamic range of the system. In Fig. 5A-C, we show that a nonisochronous oscillator compresses its response over a range that is more than an order of magnitude larger than in the isochronous case. To quantify this compression, we define the dynamic range of the system as

6 $$\begin{aligned} \gamma = \log _{10}{ \bigg [\frac{F_{10\times sync}}{F_{sync}} \bigg ]} \end{aligned}$$

where $F_{sync}$ is the minimum forcing amplitude required for the response of the system to synchronize to an external sinusoidal signal in the deterministic limit. $F_{10\times sync}$ represents the forcing amplitude required to elicit an amplitude response 10 times as large as the amplitude response at $F_{sync}$. Therefore, the dynamic range, $\gamma$, measures how many decades of forcing strength span one decade of the response amplitude. We illustrate how this metric is calculated in Fig. 5D. For quiescent systems, we define $F_{sync}$ to be the forcing required to elicit an amplitude response of 0.01. For a linear system, we would find $\gamma = 1$, while for a system that responds according to the 1/3-power-law, we would expect $\gamma = 3$.

In the deterministic limit, the dynamic range can be approximated analytically as

7 $$\begin{aligned} \gamma = 3 + \log _{10}{ \Bigg [ \frac{\mu }{|\Delta \omega |} \bigg (1 + \Big (\frac{\beta }{\alpha }\Big )^2 \bigg ) \Bigg ] } \end{aligned}$$

for $\mu > 0$ and $|\Delta \omega | > 0$. The supplementary material outlines this analytical calculation and discusses the dynamic range in the quiescent regime. We compare this approximation to numerical simulations in Fig. 5E-F. This approximation, which predicts that nonisochronicity increases the dynamic range monotonically with increasing $|\beta |$, shows good agreement with numerical data in the deterministic limit. However, in the presence of noise, this approximation breaks down for large values of $|\beta |$ and for systems near the Hopf bifurcation. In the oscillatory regime, we instead observe a peak in the dynamic range as a function of $|\beta |$, consistent with the previously discussed values of $|\beta |$ that lead to maximal sensitivity.

**Figure 6:**
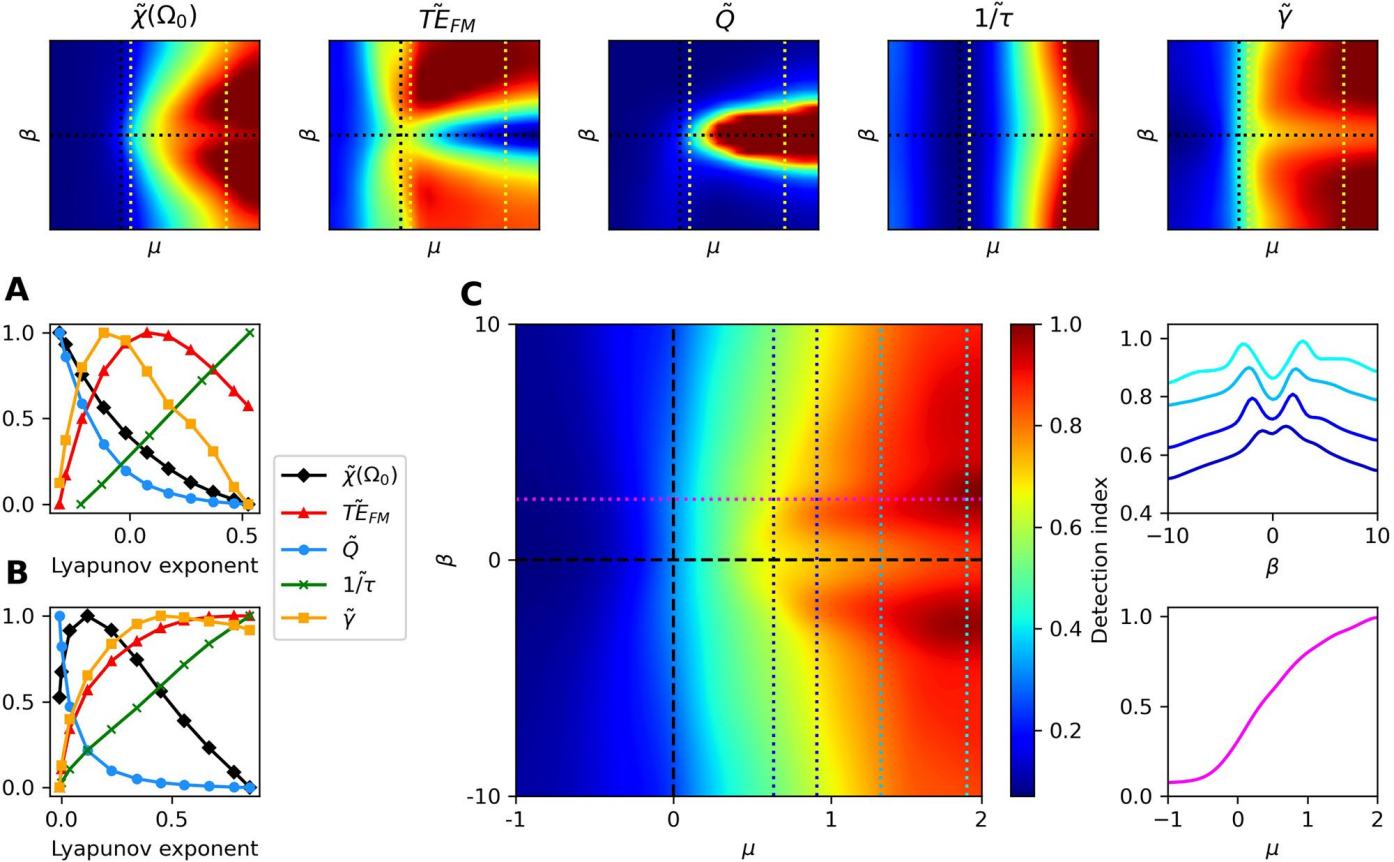
(*Narrowband detector*) The heatmaps of the five metrics of importance are shown in the top row. These metrics are plotted as functions of the level of chaos for $\mu \approx 0.14$ (**A**) and $\mu \approx 1.57$ (**B**), as indicated by the yellow, dotted lines in the top panels. (**C**) Detection index from incorporating the five measures with equal weights, $\textbf{w} = \frac{1}{5}[1, 1, 0, 1, 0, 1, 1]$. Cross-sections of the heatmap at the dotted lines are shown to the right. For all measures shown, the noise strength was set to $D=10^{-3}$, with the exception of $\tilde{Q}$, where the noise was regarded as an external stimulus. In this case, $D=0.01$.

**Figure 7:**
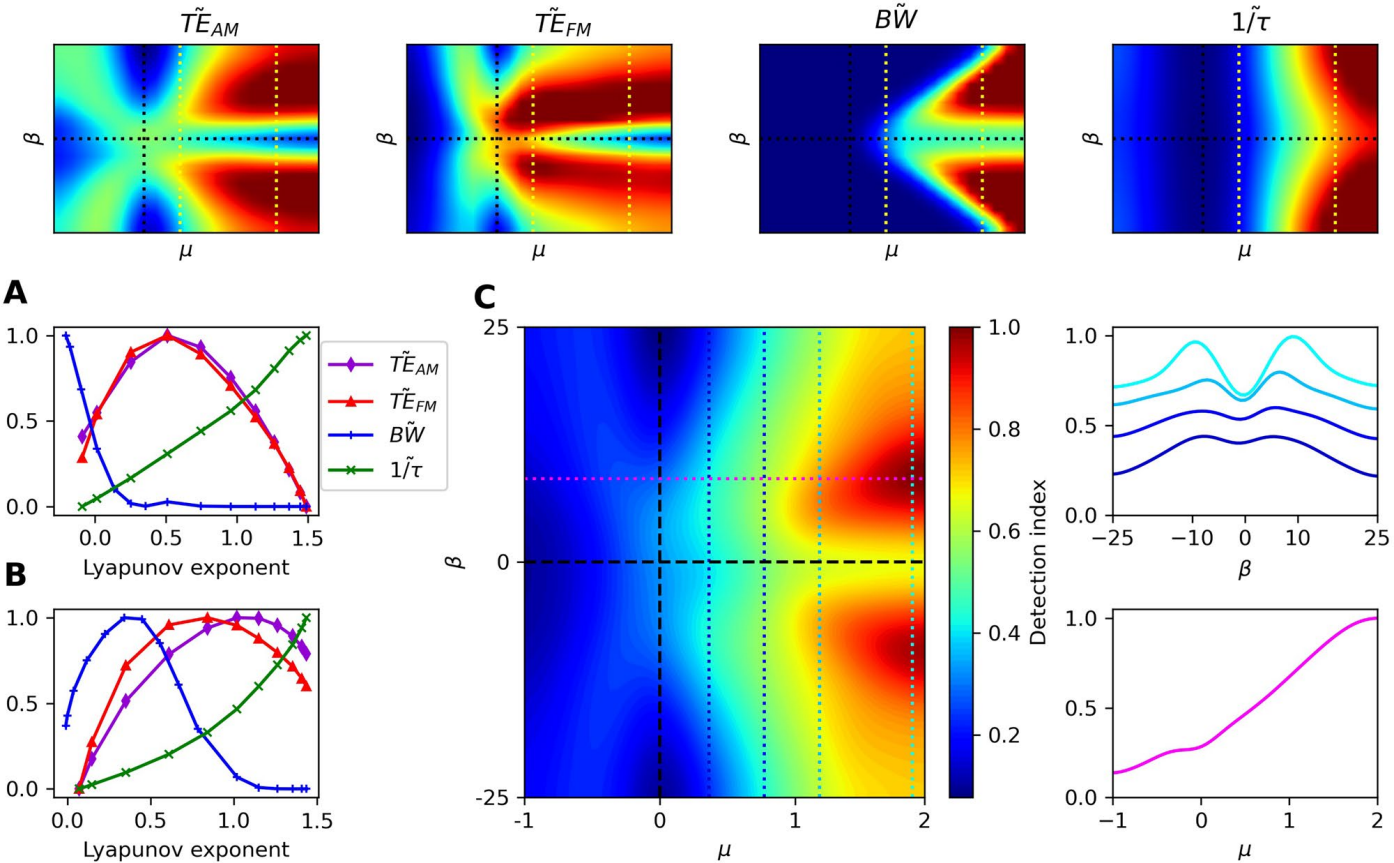
(*Broadband detector*) The heatmaps of the four metrics of importance are shown in the top row. These metrics are plotted as functions of the level of chaos for $\mu \approx 0.43$ (**A**) and $\mu \approx 1.57$ (**B**), as indicated by the yellow, dotted lines in the top panels. (**C**) Detection index from incorporating the four measures with equal weights, $\textbf{w} = \frac{1}{4}[0, 1, 1, 0, 1, 1, 0]$. Cross-sections of the heatmap at the dotted lines are shown to the right. For all measures shown, the noise strength was set to $D=10^{-3}$, with the exception of $\tilde{BW}$, where the noise was regarded as an external stimulus. In this case, $D=0.01$.

## Combining all response metrics

The best choice of parameters depends heavily on the application of the signal detector and the desired specifications. For single-frequency or narrowband detectors, it may be favorable to have the quality factor of the response as high as possible. However, for a broadband detector, a large quality factor would be unfavorable, as it would attenuate frequencies that should be captured. We note that there exists a spectrum of desired capabilities of signal detectors and no single metric can fully characterize the performance. We therefore incorporate all seven measures into a single score, which can be weighted in accordance with the application of the system. We first define the vector

8 $$\begin{aligned} \textbf{v} = \begin{bmatrix} \tilde{\chi }(\omega _0),&\tilde{TE}_{FM},&\tilde{TE}_{AM},&\tilde{Q},&\tilde{BW},&\tilde{\frac{1}{\tau }},&\tilde{\gamma } \end{bmatrix}, \end{aligned}$$

where the elements of this vector are the on-resonance linear response, transfer entropy from FM stimulus, transfer entropy from AM stimulus, quality factor, threshold bandwidth, speed of response, and dynamic range, respectively. Each element is linearly scaled such that its range runs from 0 to 1 at the minimum and maximum values in the parameter space. To better illustrate the optimal regime, we scale the measures such that they saturate at the 90th percentile of their distributions. In the supplementary material, we vary this saturation point to show that it has a negligible effect on our results (Figs. S3-S4). We then define the detection index to be the sum of the elements in $\textbf{v}$, scaled by relative weights of importance,

9 $$\begin{aligned} \text {detection index} = \textbf{w} \cdot \textbf{v}, \end{aligned}$$

where $\textbf{w}$ is a vector whose values represent the relative weights of importance of the measures.

The detection index could exhibit multiple optima, dependent on the desired specifications, or it could increase indefinitely with either $\mu$ or $\beta$, thus yielding no optimum. From the biological perspective, not only do auditory and vestibular sensors have different demands, but moreover, the same end organs from different species are likely to be optimized for different environments and various conspecific calls. Furthermore, even the same system may self tune into different regimes when placed in different acoustic surroundings. These variations can readily be captured by the appropriate selection of the weighting factor, $\textbf{w}$. We show a possible selection of weights that illustrates how a frequency-selective auditory organ may optimize its performance, and a selection of weighting factors to describe the performance of a broadband detector. In the supplementary material, we present additional weight choices for both types of detector (Figs. S3–S4).

A narrowband detector should prioritize the linear response function at resonance, the quality factor of the response, the dynamic range, the speed of response, and the transfer entropy from a narrowband FM signal. Since a large threshold bandwidth would be harmful to this detector’s purpose, we set its weight to zero and use the weight vector, $\textbf{w} = \frac{1}{5}[1, 1, 0, 1, 0, 1, 1]$. Using these weights, we find that the detection index appears to increase indefinitely with increasing $\mu$, but peaks at small $|\beta |$ when $\mu$ is fixed (Fig. 6C).

We now consider a broadband detector, where the threshold bandwidth is important instead of the quality factor. The detector should be sensitive to signal modulations and be able to detect transient signals composed of many frequencies. In this case, the information captured by signal modulations is valuable, as well as the speed of response. We therefore choose the weight vector, $\textbf{w} = \frac{1}{4}[0, 1, 1, 0, 1, 1, 0]$. The performance of this detector varies in a manner similar to the narrowband detector in that it continues to improve deeply into the oscillatory regime. However, a higher degree of nonisochronicity is preferable (Fig. 7C).

## Discussion

We have determined the performance of the Hopf oscillator as a signal detector throughout its parameter space, by varying both the proximity to criticality and the degree of nonisochronicity. To the best of our knowledge, the intersection of these two properties has not previously been studied in the context of signal detection. We first calculated the Lyapunov exponent of the system in the $\mu \beta$-plane, near the Hopf bifurcation, characterizing the level of chaos induced by noise. We showed the breakdown of a previous analytic approximation as the system approaches the Hopf bifurcation. Instead of diverging, the Lyapunov exponent decreases to zero continuously and becomes negative for $\mu < 0$, as is expected for dynamics near a fixed point. This allows us to correlate the degree of chaos with the nonisochronicity parameter, $\beta$.

Providing the oscillator with several types of stimulus, we demonstrated that the sensitivity and temporal acuity of the nonisochronous system can exceed that of the critical system, regardless of the level of external noise. Further, we showed that the nonisochronous system compresses the response of the system to large-amplitude signals, due to the increase in magnitude of the cubic parameter. This increases the dynamic range of stimulus amplitudes that the system can safely detect. All three of these measures (sensitivity, temporal acuity, and amplitude compression) have been shown to be important aspects for signal detection by the auditory and vestibular systems.

The favorable parameter regime for frequency selectivity, when assessed in isolation, depends heavily on the specific application of the detector. The tuning curves broaden as the system approaches criticality from the oscillatory side, and when the level of nonisochronicity is increased. The most sharply-tuned detectors can be found when the system is isochronous and as far into the oscillatory regime as possible. Hence, the only one of our metrics that is monotonically degraded by nonisochronicity is the quality factor. However, the speed of response, $1/\tau$, monotonically increases with increasing levels of nonisochronicity. The other five metrics are non-monotonically improved by nonisochronicity. For fixed $\mu$, each of these metrics can exhibit a peak as a function of $\beta$ or the Lyapunov exponent (Figs. 6-7).

When considering all of the measures together, the prefered parameter regime reflects the biological application of the detector and the desired specifications. The various requirements for auditory and vestibular detection have been previously explored with a numerical model of an individual hair cell bundle. In that study, the mechanical load of the system was shown to greatly influence the response metrics^42^. In the present work, we aim to understand the effects of nonisochronicity and criticality considering two scenarios. The first is a single-frequency or narrowband detector. When incorporating all of the relevant measures for this detector, we find that the preferred regime is in the oscillatory state, as far from the Hopf bifurcation as possible, and with a small amount of nonisochronicity. In this regime, the innate dynamics are weakly chaotic. We propose that this regime would be well-suited for frequency-selective auditory systems. For the second scenario, we consider a system designed to detect as broad a range of frequencies as possible, and capture information from transient stimuli, which tend to contain many frequencies. In this case, we also find that the preferred regime is as deeply into the oscillatory regime as possible, but with a large degree of nonisochronicity. We propose that this regime is well-suited for vestibular systems.

Overall, we show that all detection metrics, other than the quality factor, are enhanced by the presence of nonisochronicity. Specifically, the issue of critical slowing down is removed, while sensitivity is enhanced. Hence, this tradeoff inherent in critical systems is resolved, and evaluating different regimes of detection - broadband versus frequency selective - changes only the preferred level of $\beta$. Finally, high performance is achieved, not at a specific point, but rather in a broad range of parameter space, which would endow the biological system with flexibility and robustness, and obviate the need for extremely precise fine-tuning of parameters or the need for dynamical feedback on any of them^43^.

We note that nonisochronicity and chaos are common elements in high-dimensional dynamical systems that contain nonlinearities, regardless of the presence of stochastic processes. A previous numerical study of a deterministic Hopf oscillator found that chaos appears upon introducing a feedback equation on the control parameter^44^. We later demonstrated that this system shows improved sensitivity in this chaotic regime^20^. Chaos was also found in a 12-dimensional numerical model of hair cells^41^, and its presence was associated with enhanced compliance to step-like stimuli. As an additional demonstration, we show that the Rössler attractor^45^ is most sensitive to weak signals when poised in the weakly chaotic regime (see Supplementary Material, Figs. S5-S6). We expect chaotic dynamics to be present in other detailed models of auditory and vestibular systems, as they are both high dimensional and nonlinear.

The current study considers only single Hopf oscillators, representing individual sensory elements. While the sensory hair bundles of certain species are free-standing^46^, hair bundles of most vestibular and auditory organs studied display some degree of mechanical coupling to each other. The strength and extent of this coupling varies greatly for different specializations of the sensory organ^47^. Considering the diverse configurations of mechanical coupling, we have focused this study solely on the ability of individual Hopf oscillators to detect external signals. Understanding the joint effects of chaos and criticality on a single oscillator is important for understanding the full coupled system.

However, we point out two issues of individual detectors that have been shown to resolve in the coupled system. The first is the smearing out of the critical point in the presence of noise (Fig. S1). It has been shown that, although noise removes criticality in the individual Hopf oscillator, it can be restored in the coupled system^48^. Second, although nonisochronicity deteriorates the frequency selectivity of individual detectors, it can be restored in arrays of coupled detectors, provided that the elements synchronize to each other^49^.

Nonisochronicity is often excluded from simple numerical models of these sensory systems. It greatly increases the complexity of the system, leading to multi-stability and a chaotic response to various types of signals^19,38^, including white noise. Further, the nonisochronous term in the Hopf oscillator was shown to lead to violation of a generalized version of the fluctuation dissipation theorem, breaking any simple relations between the system’s sensitivity to stimulus and susceptibility to stochastic fluctuations^50^. In the isochronous picture, poising a system near the Hopf bifurcation can be beneficial. The transfer entropy is maximized (Fig. 2), and the system can achieve large, entrained responses to weak signals, provided that the noise is sufficiently weak. However, to make the system robust to noise, it must reside in the oscillatory regime, thereby utilizing the energy of the autonomous motion to amplify the signal. This results in a tradeoff along the control-parameter axis, leading to an optimal value for $\mu$, where the system is close enough to the bifurcation to entrain to a signal, but oscillatory enough to be robust to noise^39^.

However, when nonisochronicity is introduced, this prefered parameter choice moves deeply into the oscillatory regime, while the entrainability can then be controlled by $\beta$. This parameter can be adjusted to increase the detection capabilities of the system, with different preferred values that depend on the specific application of the system. We also note that this parameter controls the degree of synchronization in an array of coupled Hopf oscillators^51^. We therefore propose that, in addition to proximity to a critical point, nonisochronicity is an essential element in the dynamics of auditory and vestibular systems. This instability, which gives rise to chaotic dynamics, also greatly improves the performance and robustness of the Hopf oscillator as a signal detector. We speculate that nonisochronicity and chaos are important characteristics of other biological systems evolved for signal detection, as well as systems that exhibit synchronization of their active components.

## Numerical methods

Stochastic differential equations were solved using Heun’s method with time steps ranging from $2\pi \times 10^{-4}$ to $2\pi \times 10^{-3}$.

### Response amplitude and linear response function

To determine phase-locked amplitude, we first compute the average response to a sinusoidal stimulus of 64 systems, each prepared with different initial phases uniformly spaced across the deterministic limit cycle. This method ensures that any non-synchronized oscillations at the stimulus frequency will average to zero, and only signals that lock to the stimulus will be counted toward the response. We then fit a sinusoid to the mean response with frequency fixed to the stimulus frequency. We define the phase-locked amplitude as the amplitude of this fit. We then compute the linear response function by dividing this response amplitude by the forcing amplitude.

### Frequency-modulated (FM) and amplitude-modulated (AM) stimuli

The frequency-modulated forcing takes the form

10 $$\begin{aligned} F_{FM}(t) = F_0 e^{i\psi (t)}, \end{aligned}$$

where $\psi (t)$ is the instantaneous phase of the stimulus, and we set $F_0 = 0.1$. The instantaneous stimulus frequency is centered at $\Omega _0$, with additive stochastic fluctuations,

11 $$\begin{aligned} \omega (t) = \Omega _0 + \eta _f(t), \end{aligned}$$

where $\eta _f(t)$ is low-pass filtered Gaussian white noise (pink noise) with a brick-wall cutoff frequency of $\Omega _0$. We let the standard deviation of this variable equal $0.3\times \Omega _0$. We can then calculate the instantaneous phase of the stimulus,

12 $$\begin{aligned} \psi (t) = \int _0^t \omega (t') dt' = \psi (0) + \Omega _0 t + \int _0^t \eta _f(t') dt'. \end{aligned}$$

Information production is determined solely by the frequency modulator, $\eta _f(t)$. This method of generating the signal does not influence the amplitude and allows us to examine the effects of information transmission through frequency modulation alone.

Similarly, amplitude-modulated signals take the form

13 $$\begin{aligned} F_{AM}(t) = \eta _a(t) e^{i\Omega _0 t}, \end{aligned}$$

where $\eta _a(t)$ is low-pass filtered Gaussian white noise (pink noise) with a brick-wall cutoff frequency of $\Omega _0$. We set the mean and standard deviation of this stochastic amplitude modulator to be 0 and 0.5, respectively.

### Transfer entropy

The transfer entropy^40^ from process *J* to process *I* is defined as

14 $$\begin{aligned} T_{J \rightarrow I} = \sum p(i_{n+1}, i_{n}^{(k)}, j_{n}^{(l)}) \log \frac{p(i_{n+1} \ | \ i_{n}^{(k)}, j_{n}^{(l)})}{p(i_{n+1} \ | \ i_{n}^{(k)})}, \end{aligned}$$

where $i_{n}^{(k)} = (i_n,\ldots ,i_{n-k+1})$ are the *k* most recent states of process *I*. Therefore, $p(i_{n+1} \ | \ i_{n}^{(k)}, j_{n}^{(l)})$ is the conditional probability of finding process *I* in state $i_{n+1}$ at time $n+1$, given that the previous *k* states of process *I* were $i_{n}^{(k)}$ and that the previous *l* states of process *J* were $j_{n}^{(l)}$. The summation runs over all points in the time series and over all accessible states of both processes. The transfer entropy measures how much one’s ability to predict the future of process *I* is improved upon learning the history of process *J*. The measure is asymmetric upon switching *I* and *J*, as information transfer between two processes is not necessarily symmetric. For stimulus-response data, this asymmetry allows for control tests by measuring the transfer entropy from the response to the stimulus, which should be zero. The choice of *k* and *l* has little effect on the results, so we select $k = l = 5$, and sample the 5 points such that they span one mean period of the system. We discretize the signal into 4 amplitude bins, however, similar results were obtained when using 2 bins.

### Return time

We determine the return time, $\tau$, by calculating the average response from 64 simulations to a large-amplitude step stimulus,

15 $$\begin{aligned} F(t) = F_{0}[\Theta (t - t_{on}) - \Theta (t - t_{off}) ], \end{aligned}$$

where $\Theta (t)$ is the Heaviside step function, and we set $F_{0} = 5$, $t_{on} = 1$, and $t_{off} = 2$. We define the return time as the time it takes the mean response of the system to return to a value within a standard deviation of the mean steady-state amplitude, as measured by the data prior to the step onset.

### Quality factor and threshold bandwidth

To estimate the quality factor of the system response, we stimulate with additive white Gaussian noise ($D = 0.01$), and calculate the average power spectrum over 60 simulations, each with different initial conditions and realizations of noise. This produces a smooth curve, which can then be used to estimate the full width at half maximum and the quality factor of the response. We then use this curve to calculate threshold bandwidth by determining the range of frequencies for which the Fourier amplitudes exceed a threshold of 0.1. This threshold was chosen as it is approximately an order of magnitude above the noise floor of the critical system.

### Supplementary Information

Supplementary Information.

## Data Availability

The Python code used for performing the analysis and generating the figures is available online: https://github.com/jfaber3/Criticality-and-Chaos.git.
