# Supplementary material for: Criticality and chaos in auditory and vestibular sensing
Source: Sci Rep. 2024 Jun 6;14:13073. doi: 10.1038/s41598-024-63696-3 (PMC11156970; doi:10.1038/s41598-024-63696-3)
Supplement: Supplementary file 1 — Supplementary Information. [file 41598_2024_63696_MOESM1_ESM.pdf]

Supplementary Material for: Criticality and Chaos in Auditory and Vestibular Sensing

Justin Faber[1*] and Dolores Bozovic[1,2†]
[1]Department of Physics & Astronomy and [2]California NanoSystems Institute,
University of California,
Los Angeles, California 90095, USA

(Dated: April 19, 2024)

I. CRITICAL SLOWING DOWN

We calculate the time scale associated with transient solutions of the Hopf normal form equation, following a weak perturbation or stimulus. At steady-state, the stable solution to the normal form equation is $r(t) = r_0 = \sqrt{\frac{\mu}{\alpha}}$ for $\mu > 0$. We find solutions in the vicinity of this limit cycle by letting

$$r(t) = r_0 + \Delta r(t) \tag{1}$$

where $\Delta r(t)$ is the perturbation induced by the stimulus. Plugging this in and utilizing $\frac{\Delta r(t)}{r_0} \ll 1$, we find, to first order,

$$\frac{d\Delta r}{dt} = -2\mu\Delta r \tag{2}$$

which has solutions that decay with characteristic time

$$\tau = \frac{1}{2\mu} \tag{3}$$

This calculation can also be done in the quiescent regime, which yields $\tau = \frac{1}{\mu}$.

In the presence of noise, a system near the critical point becomes very susceptible to stochastic fluctuations. In Fig. S1B, we show how the amplitude of limit cycles oscillations of the system depend on the control parameter, at different levels of additive noise. As can be seen, the infinite gain that is present in the deterministic limit is removed by the presence of noise Fig. S1D, and the bifurcation point is smeared. Noise also obscures or even removes the effects of critical slowing down. We see that the long transient following a power-law decay at the critical point is shortened, as the steady-state amplitude is no longer zero (Fig. S1C). The level of noise required to obscure the effect ($D \approx 0.1$) significantly reduces the gain and smears out the bifurcation diagram.

* faber@physics.ucla.edu
† bozovic@physics.ucla.edu

II. PHASE-LOCKING RANGE

We consider sinusoidal forcing in the absence of noise ($F(t) = Fe^{i\omega t}$, $D = 0$) and express the solution in polar coordinates, $z(t) = r(t)e^{i(\omega t + \phi(t))}$, where ϕ is the phase difference between stimulus and response. The normal form equation becomes

$$\begin{aligned} \frac{dr}{dt} &= \mu r - \alpha r^3 + F\cos\phi \quad \text{and} \\ \frac{d\phi}{dt} &= \omega_0 - \omega - \beta r^2 - \frac{F\sin\phi}{r}. \end{aligned} \tag{4}$$

We consider weak forcing, where $\frac{F}{\mu r_0} \ll 1$ and $r_0 = \sqrt{\frac{\mu}{\alpha}}$. We can then assume that the stimulus only weakly perturbs the amplitude of oscillation, $r(t) = r_0 + \Delta r(t)$ where $\frac{\Delta r}{r_0} \ll 1$. Plugging this in and assuming constant Δr, we find the steady-state amplitude,

$$r_s = r_0 + \frac{F\cos\phi_s}{2\mu} \tag{5}$$

Inserting this into the phase equation leads to

$$\frac{d\phi_s}{dt} = \Omega_0 - \omega - \frac{F}{r_0}[\sin\phi_s + \frac{\beta}{\alpha}\cos\phi_s] = 0 \tag{6}$$

where $\Omega_0 = \omega_0 - \beta r_0$ is the limit-cycle frequency in the absence of stimulus. This equation has solutions for ϕ_s only when

$$\frac{F}{r_0} \geq \frac{|\omega - \Omega_0|}{\sqrt{1 + \left(\frac{\beta}{\alpha}\right)^2}}. \tag{7}$$

We have thus found the condition for synchronization to the stimulus in the absence of noise. Notice that any nonzero value for β improves this detectors ability to mode-lock to the external signal. Using this equation, we can also calculate the bandwidth over which the detector will synchronize to the signal:

$$\text{Bandwidth} = 2|\omega_{max} - \Omega_0| = \frac{2F}{r_0}\sqrt{1 + \left(\frac{\beta}{\alpha}\right)^2}, \tag{8}$$

which increases with increasing $|\beta|$.

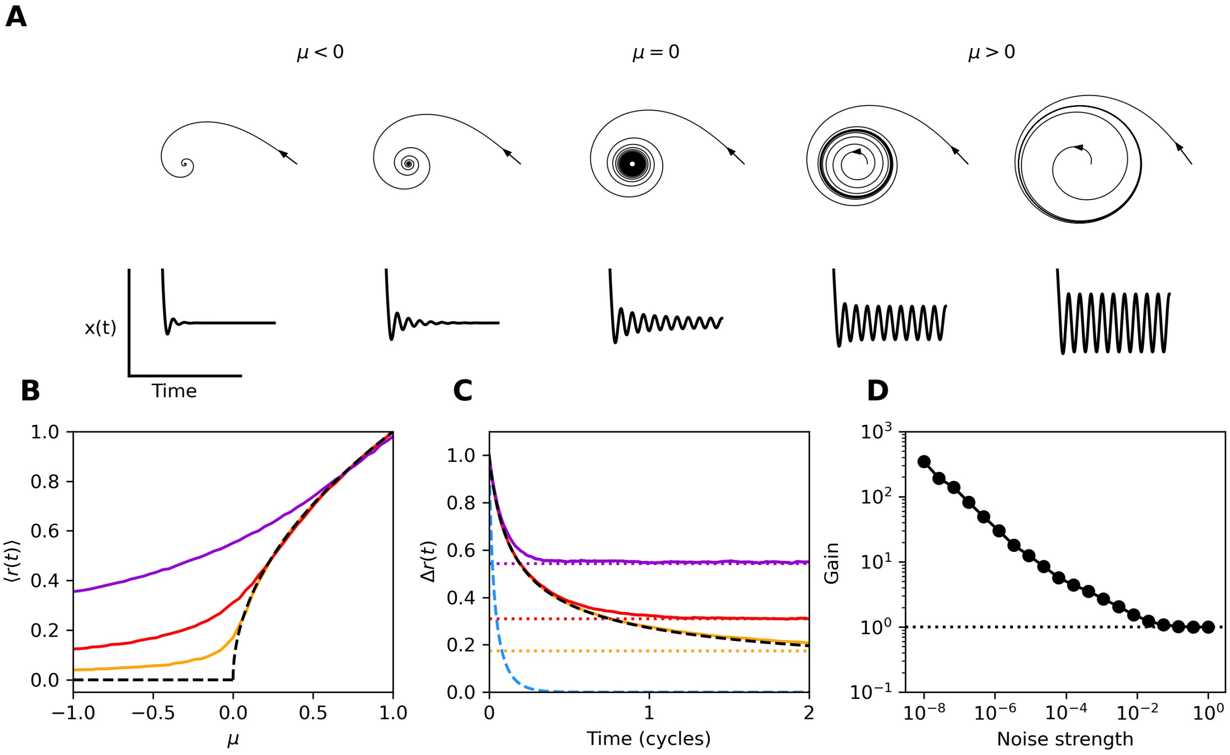

FIG. S1. (A) State-space dynamics (top row) and time traces (bottom row) of the Hopf oscillator for several values of the control parameter, increasing from left to right. (B) steady-state mean amplitude of the system near the bifurcation for several levels of additive noise. (C) Return to steady-state following a mechanical perturbation to the critical system ($\mu = 0$) averaged over many trials for several noise levels. Black, orange, red, and purple correspond to $D = 0, 0.001, 0.01,$ and 0.1, respectively. The blue, dashed curve shows a comparison to a system with $\mu = 1$ and $D = 0$. Dotted lines indicated the steady-state mean amplitude for each noise level. For the deterministic cases, this value is zero. (D) Amplitude gain at the stimulus frequency of a critical oscillator ($\mu = 0$) for various levels of noise.

III. NONLINEAR RESPONSE AMPLITUDE

Beginning with the deterministic, sinusoidally-driven Hopf oscillator,

$$\frac{dz}{dt} = (\mu + i\omega_0)z - (\alpha + i\beta)|z|^2 z + Fe^{i\omega t}, \quad (9)$$

we calculate the steady-state response at the stimulus frequency by assuming $z(t) = Re^{i(\omega t + \phi)}$, where ϕ is the phase difference between the stimulus and the response. Plugging this into the differential equation yields,

$$Fe^{-i\phi} = (\alpha R^3 - \mu R) + iR(\omega - \omega_0 + \beta R^2). \quad (10)$$

We then multiply by the complex conjugate to find,

$$F^2 = (\alpha^2 + \beta^2)R^6 + 2\big[(\omega - \omega_0)\beta - \mu\alpha\big]R^4 + \big[\mu^2 + (\omega - \omega_0)^2\big]R^2. \quad (11)$$

In the limit of large F, we keep only the leading term in R and find

$$R \approx \frac{F^{\frac{1}{3}}}{(\alpha^2 + \beta^2)^{\frac{1}{6}}}. \quad (12)$$

IV. DYNAMIC RANGE

Using Eq. 7, we know that the minimum forcing amplitude required to entrain the response of an active Hopf oscillator ($\mu > 0$) in the absence of noise is

$$F_{sync} = \frac{|\Delta\omega| r_0}{\sqrt{1 + \left(\frac{\beta}{\alpha}\right)^2}}, \quad (13)$$

where $\Delta\omega = \omega - \Omega_0$. Using Eq. 12, we can approximate the forcing required to elicit a response amplitude of $10 \times r_0$ to be

$$F_{10\times sync} \approx 1000 r_0^3 \sqrt{\alpha^2 + \beta^2}. \quad (14)$$

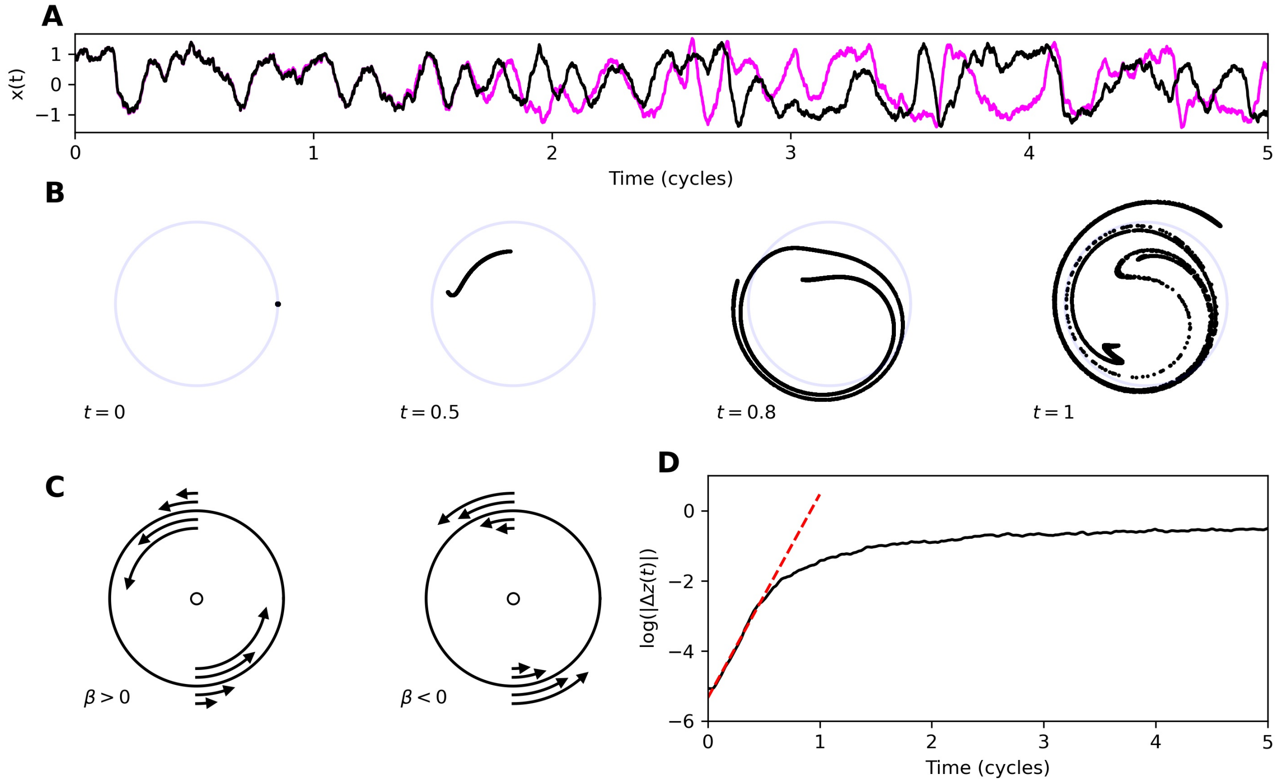

FIG. S2. (A) Two diverging time series traces of the nonisochronous Hopf oscillator given identical realizations of noise but slightly different initial conditions. (B) Snapshots at four points in time of 10^4 systems prepared all with slightly different initial conditions. The circles illustrate the location of the limit cycle in the absence of noise. (C) Illustrations of the phase space vector field of nonisochronous oscillators. Angular velocity (frequency) is dependent on the radius (amplitude) of oscillation. (D) Natural log of the average difference between pairs of trajectories, averaged over many realizations of noise. The initial linear growth corresponds to exponential divergence of trajectories. The slope of the best-fit line to the initial divergence (red-dashed) corresponds to the Lyapunov exponent.

Combining these two expressions, and using the definition in the main text,

$$\gamma = \log_{10}\left[\frac{F_{10\times sync}}{F_{sync}}\right], \tag{15}$$

we find,

$$\gamma \approx 3 + \log_{10}\left[\frac{\mu}{|\Delta\omega|}\left(1 + \left(\frac{\beta}{\alpha}\right)^2\right)\right], \tag{16}$$

for $\mu > 0$ and $|\Delta\omega| > 0$. Similarly in the quiescent regime ($\mu < 0$), we find

$$\gamma_q \approx 3 + \log_{10}\left(R_{sync}^2\sqrt{\frac{\alpha^2+\beta^2}{\mu^2+(\Delta\omega)^2}}\right), \tag{17}$$

where R_{sync} is an arbitrary small amplitude of response in the quiescent regime. We set $R_{sync} = 0.01$ and increase the forcing until the response increases 4 decades. This ensures validity of both the small and large R approximations.

V. NONISOCHRONICITY AND CHAOS

To distinguish exponentially diverging, chaotic trajectories from simple diffusion induced by noise, it is useful to introduce identical realizations of noise (common noise) to systems with slightly different initial conditions (Fig. S2A-B). If the distance between neighboring trajectories diminishes with time or remains the same, the system is non-chaotic. However, if the common noise stimulus causes neighboring trajectories to diverge exponentially, then the system exhibits noise-induced chaos [1, 2]. We note that the exponential divergence is observed only over short time scales; as a chaotic system is bounded in phase space, the distance between trajectories plateaus with time (Fig. S2D).

VI. DETECTION INDEX

In this section, we provide the detection index heatmaps for various weight choices and saturation points. Each measure is scaled by its saturation point and everything greater than or equal to this value is set to 1. The various saturation points are indicated in the histograms (Figs. S3-S4).

VII. RÖSSLER ATTRACTOR

To disentangle the effects of nonisochronicity and chaos, we explore here a 3-dimensional deterministic system that exhibits a chaotic regime. We demonstrate that the Rössler attractor [3] is most sensitive to weak, sinusoidal stimulus when poised in the weakly chaotic regime. The forced Rössler attractor is described by three differential equations,

$$\begin{aligned} \frac{dx}{dt} &= -y - z + F\cos(\omega t) \\ \frac{dy}{dt} &= x + ay + F\sin(\omega t) \\ \frac{dz}{dt} &= b + z(x - c), \end{aligned} \tag{18}$$

where F and ω represent the amplitude and frequency of the external signal. We fix $b = 0.2$ and $c = 5.7$, and vary a between 0.07 and 0.3 to control the level of chaos. We measure the Lyapunov exponent in the absence of stimulus by tracking the divergence rate of pairs of trajectories. We then apply a weak, sinusoidal stimulus to 100 identical systems, all prepared with different initial conditions. subsequently, we calculate the linear response function of the mean trajectory, as described in the main text. After repeating this process over a range of stimulus frequencies that spans the dominant frequencies of the system, we find that the sensitivity increases as the system approaches the chaotic regime. The sensitivity is largest in the weakly chaotic regime and near the onset of chaos S5).

Lastly, we show that this effect does not depend on nonisochronicity. To characterize the level of nonisochronicity in this 3-dimensional system, we track the instantaneous velocity, $\vec{v} = [\dot{x}, \dot{y}, \dot{z}]$ throughout the phase space, in the absence of stimulus ($F = 0$). We define the generalized angular frequency as

$$\dot{\Theta} = \frac{\sqrt{|\vec{v}|^2 - (\frac{dr}{dt})^2}}{r}, \tag{19}$$

where $r = \sqrt{x^2 + y^2 + z^2}$ is the instantaneous amplitude, and the numerator represents the velocity component orthogonal to $\vec{r} = [x, y, z]$. We calculate this angular frequency throughout long phase-space trajectories and determine its dependence on the instantaneous amplitude.

We plot $\dot{\Theta}$ as a function of r (Fig. S6A), showing that the instantaneous frequency has little dependence on amplitude. To quantify this dependence, we fit the data points to a function of the form $\dot{\Theta} = \beta_{fit} \times r^{p_{fit}}$. These two fit parameters characterize the level of nonisochronicity of the system, with larger magnitudes indicating higher levels of nonisochronicity. A perfectly isochronous system would yield $p_{fit} = 0$. Note that for a Hopf oscillator, we should expect $\beta_{fit} \approx \beta$ and $p_{fit} \approx 2$ (see Eq. 4 of the main text). Surprisingly, we find that the nonisochronicity of the Rössler attractor is reduced as the system enters the chaotic regime. Moreover, in this regime, the system is nearly isochronous, with $|p_{fit}| \ll 1$ (Fig. S6B-C). Hence, in a system that exhibits chaos without the necessity of stochastic noise or nonisochronicity, sensitivity is likewise improved in the weakly chaotic regime and near the onset of chaos.

[1] D. S. Goldobin and A. Pikovsky, Synchronization and desynchronization of self-sustained oscillators by common noise, Physical Review E **71**, 15 (2005).

[2] A. B. Neiman, K. Dierkes, B. Lindner, L. Han, and A. L. Shilnikov, Spontaneous voltage oscillations and response dynamics of a Hodgkin-Huxley type model of sensory hair cells, The Journal of Mathematical Neuroscience **1**, 11 (2011).

[3] O. E. Rössler, An equation for continuous chaos, Physics Letters A **57**, 397 (1976).

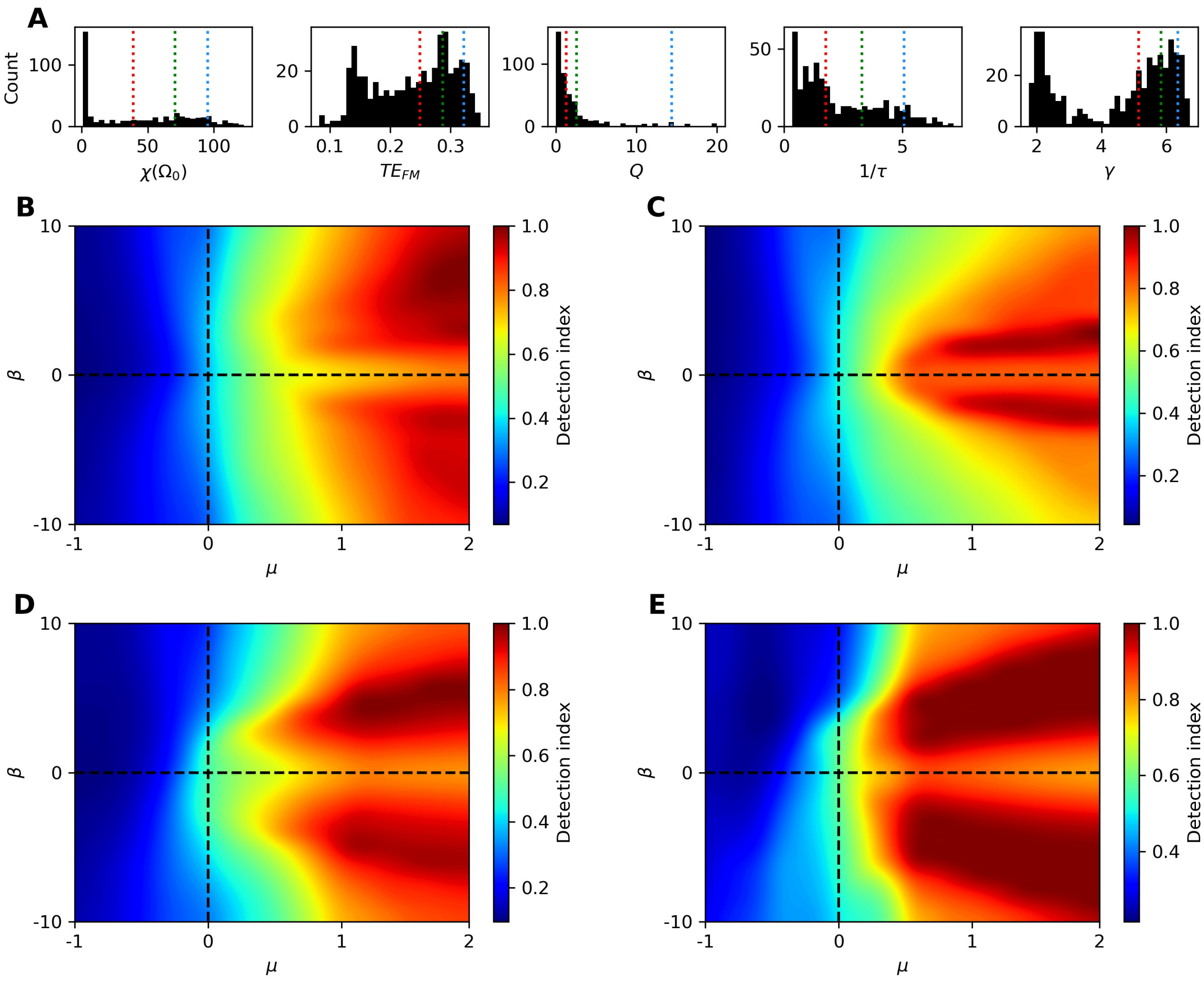

FIG. S3. (*Narrowband Detector*) (A) Histograms of all values of the individual response metrics. The saturation points are indicated by the vertical dotted lines (90th percentile in blue, 70th percentile in green, and 50th percentile in red). (B) Detection index for a system that prioritizes sensitivity with $\mathbf{w} = \frac{1}{7}[2, 2, 0, 1, 0, 1, 1]$ and a 90th-percentile saturation point. (C) Detection index for a system that equally prioritizes only sensitivity and quality factor with $\mathbf{w} = \frac{1}{3}[1, 1, 0, 1, 0, 0, 0]$ and a 90th-percentile saturation point. (D) Detection index for $\mathbf{w} = \frac{1}{5}[1, 1, 0, 1, 0, 1, 1]$ and a 70th-percentile saturation point. (E) Detection index for $\mathbf{w} = \frac{1}{5}[1, 1, 0, 1, 0, 1, 1]$ and a 50th-percentile saturation point.

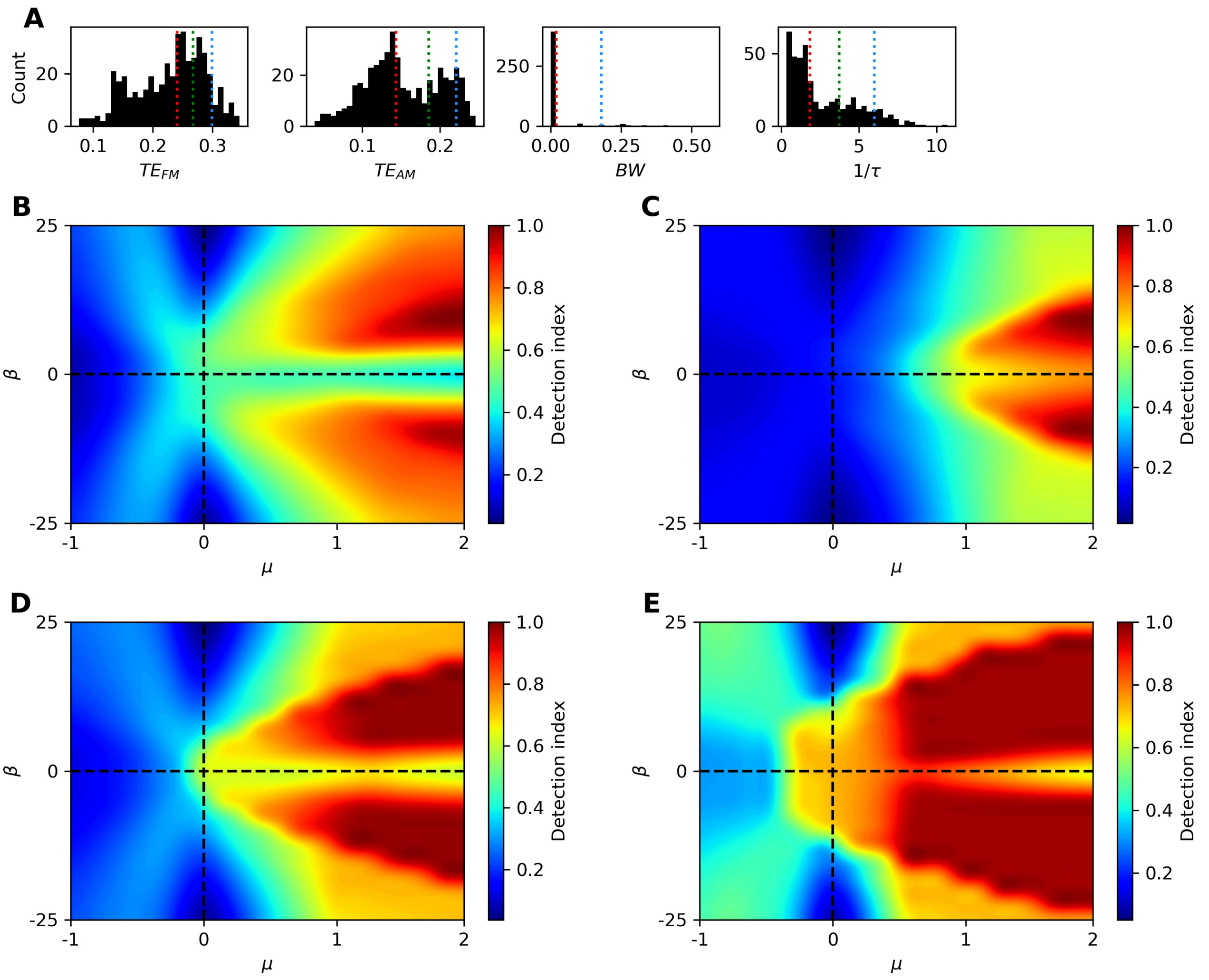

FIG. S4. (*Broadband Detector*) (A) Histograms of all values of the individual response metrics. The saturation points are indicated by the vertical dotted lines (90th percentile in blue, 70th percentile in green, and 50th percentile in red). Note that for the threshold bandwidth, many elements are zero. If the saturation point chosen is zero, we simply take any values above zero to be 1. (B) Detection index for a system that prioritizes information transfer with $\mathbf{w} = \frac{1}{8}[0, 3, 3, 0, 1, 1, 0]$ and a 90th-percentile saturation point. (C) Detection index for a system that prioritizes bandwidth of detection and temporal acuity with $\mathbf{w} = \frac{1}{8}[0, 1, 1, 0, 3, 3, 0]$ and a 90th-percentile saturation point. (D) Detection index for $\mathbf{w} = \frac{1}{4}[0, 1, 1, 0, 1, 1, 0]$ and a 70th-percentile saturation point. (E) Detection index for $\mathbf{w} = \frac{1}{4}[0, 1, 1, 0, 1, 1, 0]$ and a 50th-percentile saturation point.

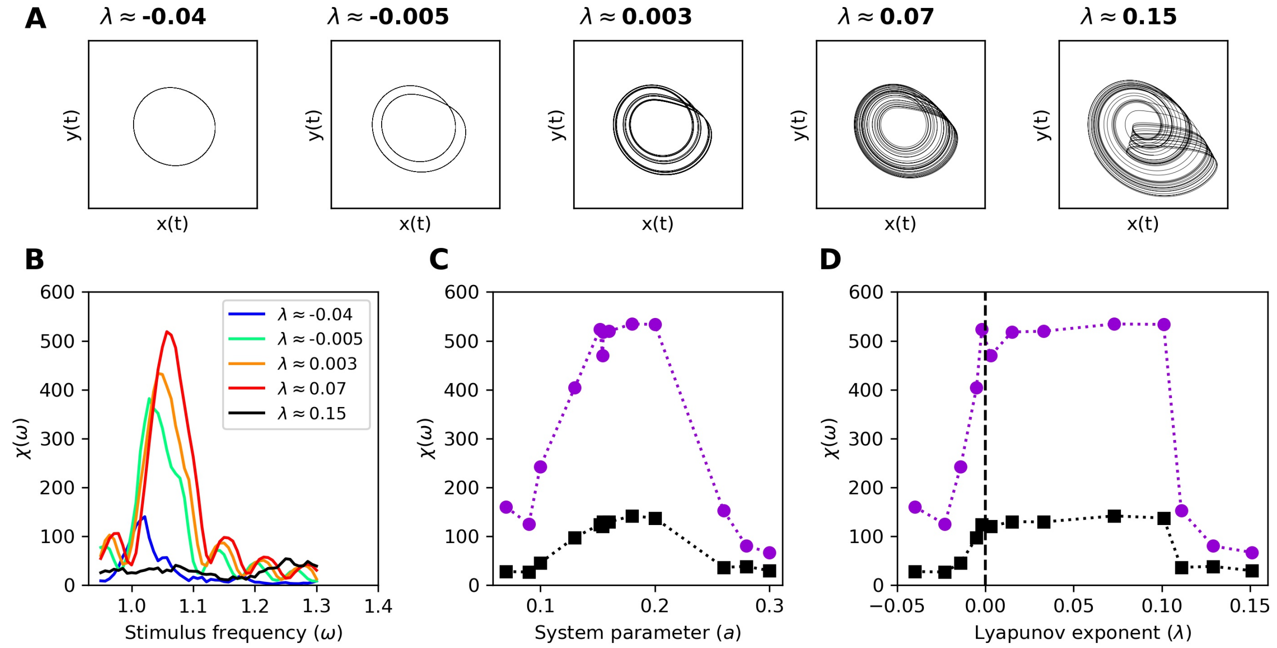

FIG. S5. (*Rössler Attractor*) (A) Phase-space plots of the Rössler attractor in the absence of stimulus for several values of the system parameter, a. Lyapunov exponents are indicated above each panel. (B) Linear response functions of these five systems in response to weak forcing ($F = 0.01$). (C-D) Maximum values (purple circles) and mean values (black squares) of the response functions over a range of stimulus frequencies ($\omega = 0.95$ to $\omega = 1.3$). The vertical dashed line in (D) indicates the border between non-chaotic ($\lambda < 0$) and chaotic ($\lambda > 0$) systems.

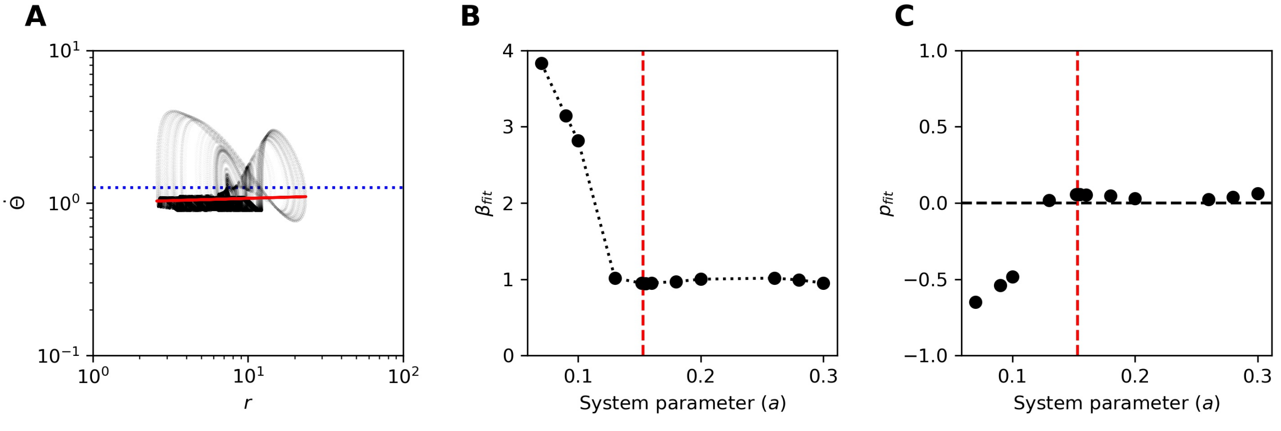

FIG. S6. (*Rössler Attractor*) (A) Example of the power-law fit (red curve) relating the instantaneous frequency to instantaneous amplitude, for 10^5 time steps and $a = 0.2$. 90% of the data points lie below the blue dotted line. (B-C) Dependence of the fit parameters on system parameter, a. Red, dashed vertical lines indicate the boundary between non-chaotic ($a < 0.153$) and chaotic ($a > 0.153$) systems. A perfectly isochronous system would have $p_{fit} = 0$, as indicated by the horizontal dashed line in (C).
